# Utilizing ultrasound in suspected necrotizing enterocolitis with equivocal radiographic findings

**DOI:** 10.1186/s12887-023-03932-3

**Published:** 2023-03-24

**Authors:** Michelle P. Kallis, Bailey Roberts, Danielle Aronowitz, Yan Shi, Aaron M. Lipskar, John B. Amodio, Alpna Aggarwal, Chethan Sathya

**Affiliations:** 1 Northwell Health North Shore/Long Island Jewish General Surgery, 300 Community Drive, Manhasset, NY 11030 USA; 2 Donald and Barbara Zucker School of Medicine at Hofstra/Northwell, 500 Hofstra Blvd, Hempstead, NY 11549 USA; 3 Division of Pediatric Surgery, Cohen Children’s Medical Center, 1111 Marcus Avenue, Suite M15, New Hyde Park, NY 11042 USA; 4 Department of Surgery, Division of Pediatric Surgery, Texas Children’s Hospital, Baylor College of Medicine, 6701 Fannin Street, Houston, TX 77030 USA; 5 Department of Radiology, Cohen Children’s Medical Center, 269-01 76th Avenue, New Hyde Park, NY 11040 USA; 6 Department of Neonatology, Cohen Children’s Medical Center, 269-01 76th Avenue, New Hyde Park, NY 11040 USA

**Keywords:** Neonatology, Antibiotic use, Pediatric surgery, Necrotizing enterocolitis, Ultrasound

## Abstract

**Background:**

To examine the use of abdominal ultrasound (AUS) as a diagnostic adjunct in the diagnosis of necrotizing enterocolitis (NEC) in cases where abdominal radiography (AXR) is equivocal in order to reduce unnecessary antibiotic use in neonates.

**Methods:**

Retrospective study (2017–2019) of infants undergoing NEC evaluation with equivocal AXR findings (*n* = 54). Paired AXR and AUS were reviewed with respect to presence or absence of pneumatosis. Concordance of AUS findings with decision to treat for NEC was evaluated.

**Results:**

Among 54 infants where AXR was equivocal, AUS demonstrated presence of pneumatosis in 22 patients (41%), absence of pneumatosis in 31 patients (57%), and was equivocal in 1 patient. All patients with pneumatosis on AUS were treated for NEC. Of 31 patients without pneumatosis on AUS, 25 patients (78%) were not treated for NEC. Patients without pneumatosis on AUS received a significantly shorter mean duration of antibiotics compared to those with pneumatosis (3.3 days (+/− 4.8 days) vs 12.4 days (+/− 4.7 days)); *p* < 0.001). Of those patients not treated, none required treatment within 1 week following negative AUS.

**Conclusion:**

AUS is a valuable tool for evaluating the presence or absence of pneumatosis in the setting of equivocal AXR. Absence of pneumatosis on AUS informs clinical decision making and reduces unnecessary treatment and antibiotic usage.

## Introduction

Necrotizing enterocolitis (NEC) is an inflammatory enterocolitis common in premature infants that is diagnosed by an array of clinical and radiographic findings. The diagnosis is often challenging and there are few definitive tests or biomarkers. The presence or absence of pneumatosis is one of the most objective and reproducible findings in severe NEC. As a result, abdominal radiography (AXR) has long been considered the radiographic gold standard for confirming the diagnosis and determining the severity of NEC, and for following the progression of disease [1]. Classically, gas-forming bacteria invade the intestinal mucosa in patients with NEC and generate intramural gas to produce the finding of pneumatosis intestinalis and portal venous air on AXR [2]. These findings are pathognomonic for NEC based on the Modified Bell’s Staging Criteria, and if diagnosed early can be medically managed with bowel rest and antibiotics. Early diagnosis and management of NEC can potentially prevent progression towards bowel perforation and avoid need for surgical intervention [3]. However, it is well known that the capability of AXR to identify pneumatosis, portal venous gas, or even intraperitoneal free air is limited [4–6].

AXR findings in neonates undergoing evaluation for NEC are often described as non-diagnostic or equivocal, even by our most experienced pediatric radiologists [4, 7]. In many instances, it is difficult to differentiate between pneumatosis versus intraluminal stool using AXR, often leading to a significant diagnostic dilemma. Lack of a definitive diagnosis may result in significant morbidity and mortality in unrecognized cases, or unnecessary treatment with broad spectrum antibiotics and parenteral nutrition in indeterminate cases. Abdominal ultrasound (AUS) is being used in some centers as a portable and non-invasive diagnostic adjunct when AXR is equivocal to provide real-time assessment of bowel wall perfusion and function; additionally, AUS may have increased sensitivity for detection of pneumatosis and portal venous gas [8, 9].

Despite studies demonstrating the potential benefits of using AUS in both establishing a diagnosis and determining the prognosis of NEC [1, 4, 8, 10–13], the widespread use of AUS remains limited by resource availability, technical skill and comfort of radiologists and sonographers, as well as unfamiliarity of clinicians regarding how to interpret the data provided by AUS. Given these limitations, there is hesitation amongst a majority of neonatologists to use the results of AUS to guide treatment [14]. The slow integration of AUS to diagnose NEC may include poor diagnostic consensus by neonatologists, surgeons, and radiologists, high degree of operator variability, lack of standardized algorithms that include AUS, and knowledge gaps in the literature [14].

The objective of this study was to determine if AUS could demonstrate the presence or absence of pneumatosis in patients with equivocal AXR findings being evaluated for NEC and thereby impact treatment decisions, with specific focus on reducing unnecessary antibiotic usage in these neonates.

## Materials and methods

This single center retrospective review, was performed at our 57 bed, Level 4 Neonatal Intensive Care Unit (NICU), and included neonates cared for between May 2017 to October 2019. Cohen Children’s Medical Center is an academic free standing quaternary care children’s hospital and regional perinatal center. A database maintained by the pediatric radiology department was reviewed, which included all patients who had AUS performed to evaluate the presence or absence of pneumatosis following an equivocal AXR obtained to evaluate for NEC. Language within equivocal AXR reports included statements such as “cannot exclude pneumatosis”, “questionable for pneumatosis”, “may represent pneumatosis” or “concerning for pneumatosis”. Several AXR reports also noted that “evaluation for pneumatosis was limited due to presence of large stool burden or bowel distension”.

Exclusion criteria included age ≥ 180 days or evidence of frank bowel perforation at time of suspicion for NEC. Approval was obtained from the institutional review board prior to collection of data (IRB# 20-0127). Collected data were managed using REDCap (Research Electronic Data Capture) [15, 16], a secure web-based research database. Demographic information included sex, reported race, birth weight, gestational age at birth, and age at AUS. Clinical data collected on day of AUS included weight, blood pressure, heart rate, highest recorded temperature, ventilatory status, and reported comorbidities. When present, laboratory values captured included CBC, coagulation studies, arterial blood gas, and CRP levels. Presenting symptoms assessed included abdominal distension, blood per rectum, bilious emesis, diarrhea, difficulty feeding, and lethargy. Other symptoms associated with patient presentation as noted in the medical record by neonatologist or pediatric surgeon were also collected.

All imaging was performed by trained sonographers and read by attending pediatric radiologists with experience in performing and reading AUS for the evaluation of NEC. The AUS protocol used at our institution for evaluating for NEC begins with scanning all four quadrants of the abdomen (RLQ, RUQ, LUQ, and LLQ). Images were obtained in sagittal and transverse views. The bowel within in each quadrant was examined. If the bowel wall could not be visualized the study was terminated. The bowel was assessed for thickness, echogenicity, peristalsis, and pneumatosis. The portal vein was also assessed for presence of portal venous gas. Ascites, simple or complex, was noted when present.

In cases where multiple AXRs were obtained, the initial AXR done on the day of suspicion for NEC, as identified by documentation in the electronic medical record, was considered for analysis. The first AUS obtained following the initial AXR was reviewed for presence or absence of pneumatosis. Patients were defined as “Treated for NEC” if antibiotics were initiated and subsequently continued for a full course, or “Not treated for NEC” if antibiotics were not started or discontinued after initiation due to imaging demonstrating the absence of pneumatosis. A full course of antibiotics was defined as an antibiotic course of a minimum of 7-14 days with intent to treat a diagnosis of NEC by the clinical team. This determination was based on assessment by the neonatology and pediatric surgery teams, as documented in the electronic medical record. In addition to radiologic findings, this decision was made based on each patient’s overall clinical picture including presenting symptoms, laboratory values, and vital signs. Concordance of radiographic findings and decisions to treat for NEC based on AUS were evaluated.

Statistical analysis was conducted using two-tailed Student’s t-test. Continuous variables were reported as means (+/− standard deviations). The sensitivity, specificity, positive predictive value, and negative predictive value of AUS to diagnose NEC based on presence or absence of pneumatosis were calculated with their 95% confidence intervals (95% CI). *P* values ≤0.05 were considered statistically significant. All statistical tests and figure graphics were prepared using GraphPad Prism 8 (version 8.4.2) software.

## Results

A total of 54 patients being evaluated for suspected NEC with equivocal AXR findings were identified within the study period. Of these 54 patients, 34 (63%) were male. Mean age at time of suspicion for NEC was 35.3 days (+/− 33.7 days). Mean gestational age at birth for our study population was 29.7 weeks (+/− 5.32 weeks). Forty-six patients (85%) had birth weight recorded within the medical record. The 8 patients where no birth weight was available were born at outside institutions and transferred for care. Amongst patients where birth weight was available, mean birth weight was 1.52 kg (+/− 1.09 kg). The most common comorbidity in our patient population was prematurity with 42 patients (78%) born before 36 weeks gestational age, and 23 of these patients (43%) born before 28 weeks gestational age. Twenty-six patients (48%) required mechanical ventilation at the time of evaluation for NEC. Eight patients (15%) had documented cardiac abnormalities including 3 patients with ventricular septal defect, 2 patients with double outlet right ventricle, 1 patient with anomalous pulmonary venous return, 1 patient with an atrioventricular canal defect, and 1 patient with coarctation of the aorta. Other common comorbidities included sequela typically associated with prematurity including 6 patients (11%) with patent ductus arteriosus and 6 patients (11%) with intraventricular hemorrhage of any grade. Patient demographics are depicted in Table 1.

**Table 1:** Patient demographics

*N*=	54
Gestational age at birth, mean (range), weeks	29.7 (23.0–40.0)
Age at time of AUS, mean (range), days	35.3 (1–159)
Birth weight, mean (range) kg	1.52 (0.45–4.5)
Weight at time of AUS, mean (range), kg	2.0 (0.5–5.50)
Male sex n(%)	34 (63)
Race n(%)	
White	12 (22.2)
African-American	18 (33.3)
Asian	7 (13)
More than one race/unknown	17 (31.5)
Comorbidities n(%)	50 (92.6)
Prematurity	42 (77.8)
Patent ductus arteriosus	6 (11.1)
Intraventricular hemorrhage	6 (11.1)
Cardiac abnormality	8 (14.8)
Mechanical Ventilation	26 (48.1)

**Fig. 1:**
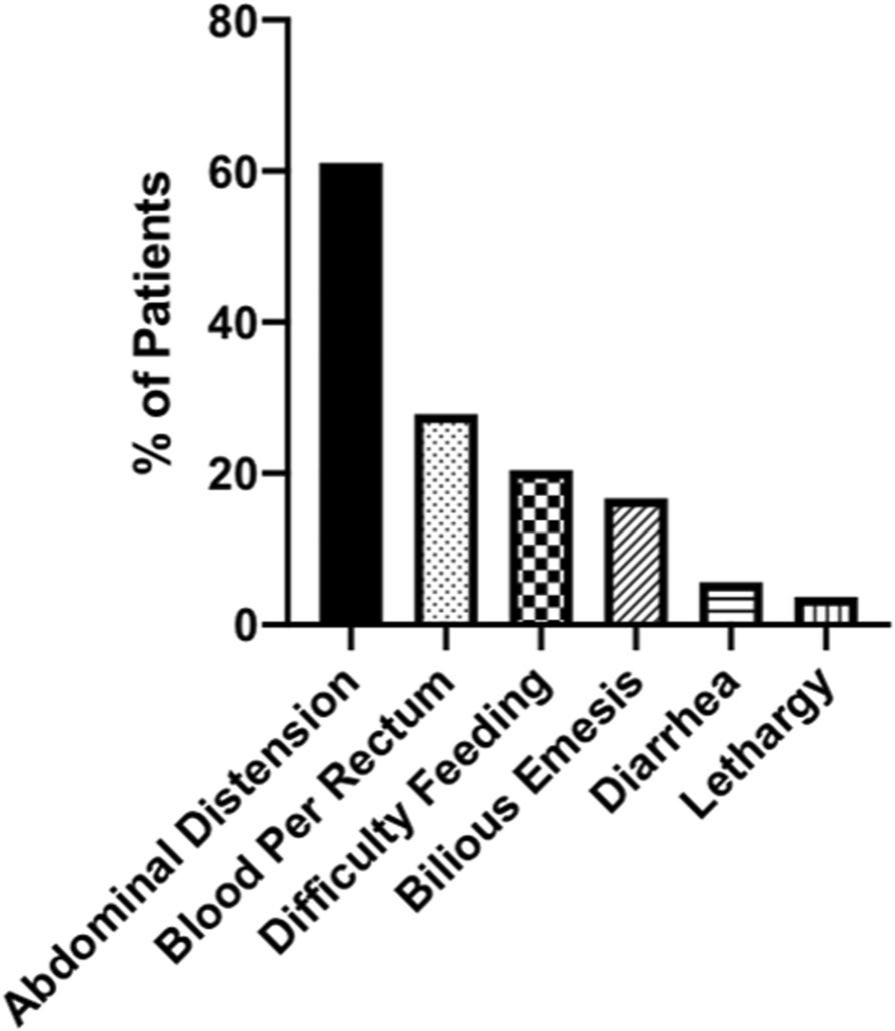
Clinical Presenting Symptoms

Common presenting symptoms in our patient cohort are depicted in Fig. 1. Thirty-three patients (66%) had abdominal distension, 15 patients (28%) had blood per rectum, and 11 patients (20%) had difficulty feeding noted in the medical record preceding evaluation for NEC.

**Fig. 2:**
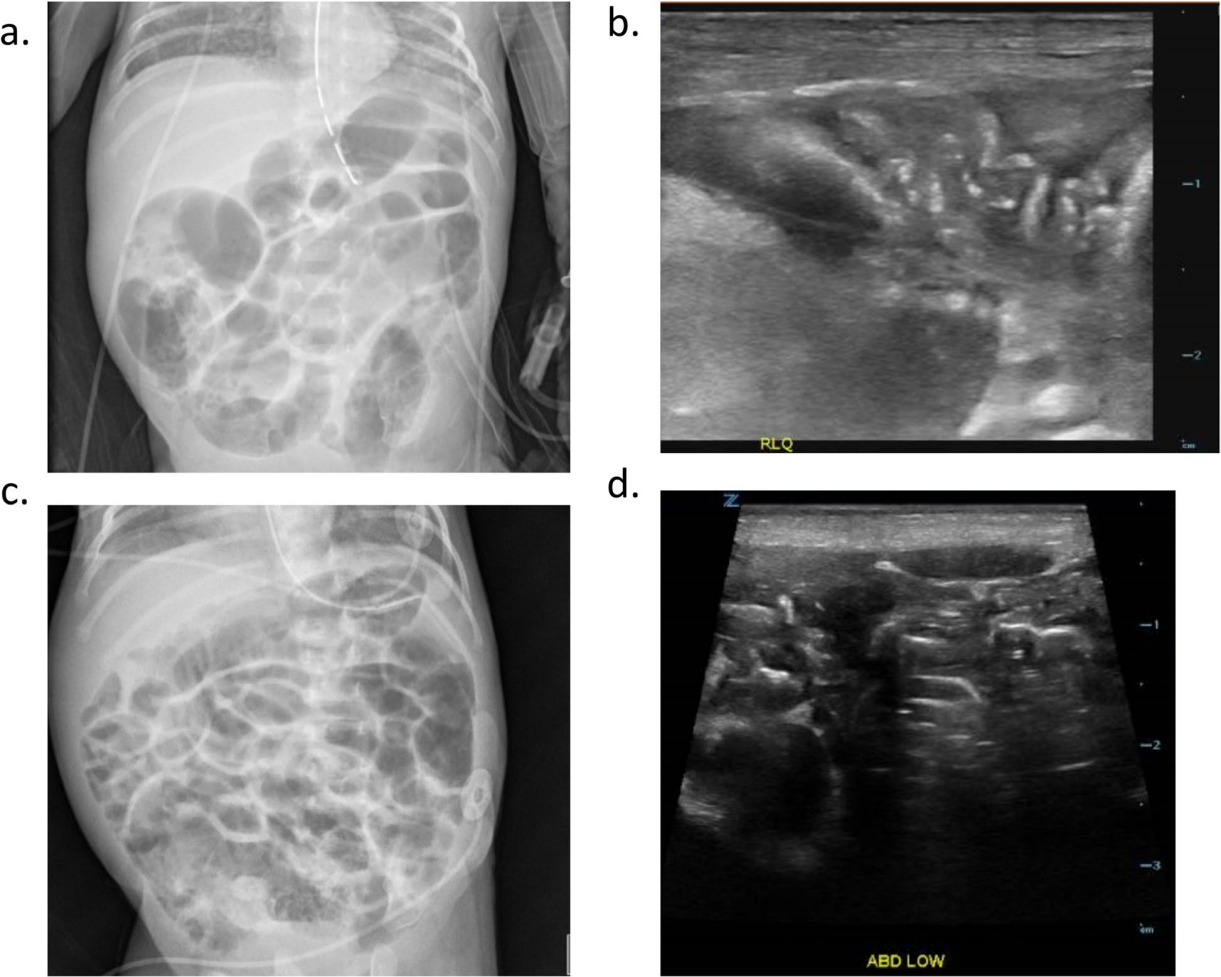
Example of Equivocal AXR with Corresponding AUS. **a**, **b** Example of equivocal AXR with corresponding AUS demonstrating pneumatosis. **a** Equivocal AXR where “bubbly lucencies” were noted in right lower quadrant and were read as “may represent pneumatosis”. **b** Corresponding AUS in the same patient. Images of the right abdomen depict bowel with “thickened wall” and “scattered increased areas of hyperechogenicity”, “findings consistent with pneumatosis”. **c**, **d** Example of equivocal AXR with corresponding AUS demonstrating absence of pneumatosis. **c** Equivocal AXR where “bubbly lucencies” were noted in the lower abdomen and could not be definitively differentiated from stool. **d** Corresponding AUS in the same patient. Images of the lower abdomen were read as “no pneumatosis”

Following equivocal AXR, average time to obtaining AUS was 7.6 h (+/− 5.6 h). Examples of equivocal AXR with corresponding AUS are depicted in Fig. 2. AUS demonstrated presence of pneumatosis in 22 patients (41%) and absence of pneumatosis in 31 patients (57%). One patient was found to have equivocal AUS findings. All 22 patients with findings of pneumatosis on AUS were treated for NEC. Of patients with pneumatosis noted on AUS, 17 patients (77%) had additional findings concerning NEC. The most common additional AUS findings in these patients included free fluid in 12 patients (55%), thickened bowel wall in 9 patients (41%), and portal venous gas in 5 patients (23%).

**Fig. 3:**
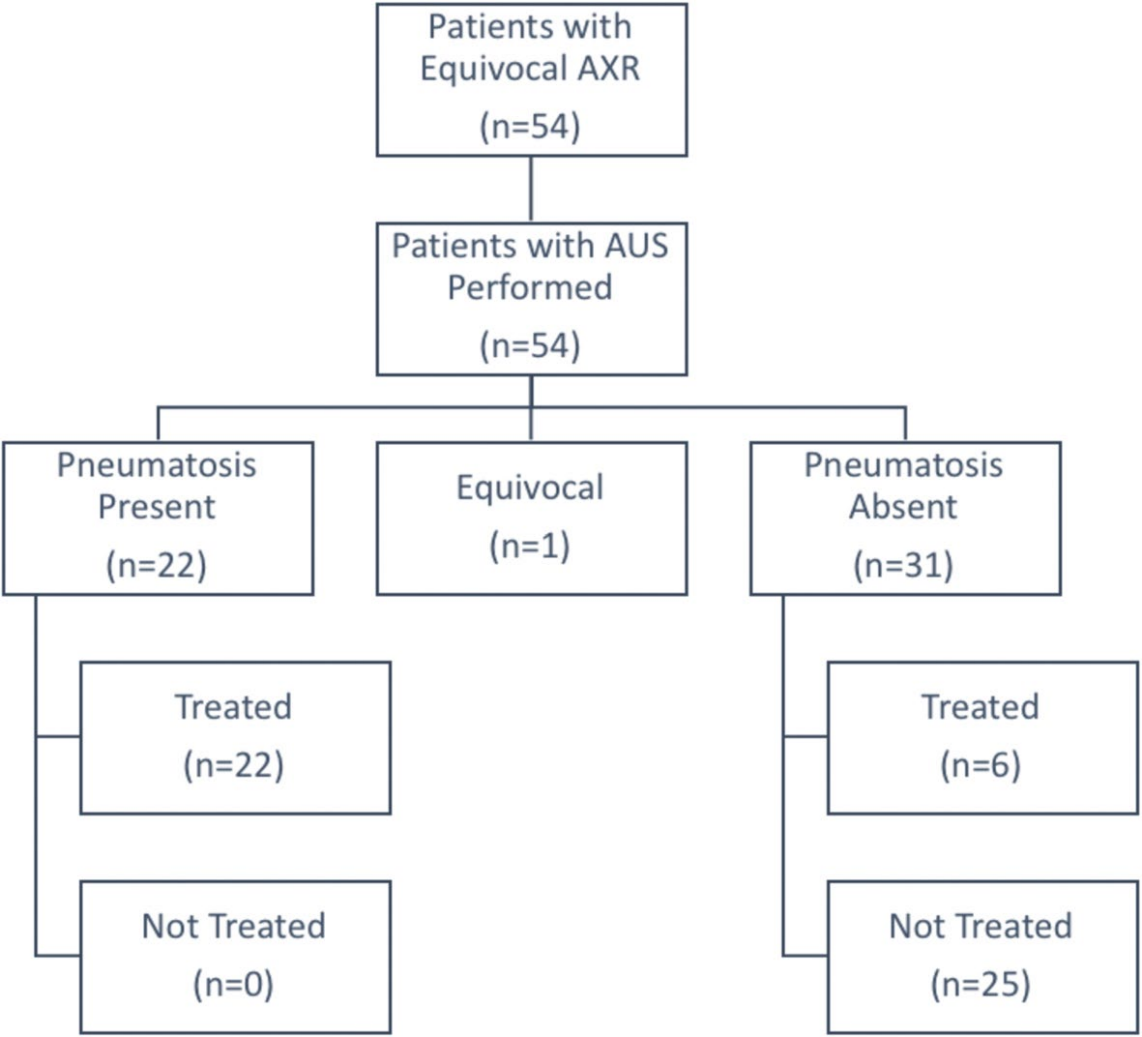
AUS Results and Resultant Treatment Decisions

Of the 31 patients without pneumatosis on AUS, 25 patients (78%) were not treated for NEC. The remaining 6 patients without pneumatosis on AUS plus the 1 patient with equivocal AUS were treated for NEC based on clinical criteria (Fig. 3). Sensitivity was 78.6% (22 of 28 patients; 95% CI: 60.5 to 89.8%) and specificity was 100% (25 of 25 patients; 95% CI: 86.7 to 100%). The positive predictive value 100% (22 of 22 patients; 95% CI: 85.1 to 100%) and the negative predictive value was 80.6% (25 of 31 patients; 95% CI: 63.7 to 90.8%).

The most common reasons cited in the medical record for treating patients for NEC despite lack of pneumatosis on AUS were persistence of symptoms and clinical deterioration. Although AUS did not show definitive pneumatosis, 5 of the 7 patients (71%) had abnormal AUS findings. Specifically, 4 patients were found to have free fluid in the abdomen and 1 patient was noted to have segments of thinned-walled bowel. Reasons cited in the electronic medical record for continuing treatment for NEC in these 7 patients are described in Table 2.

**Table 2:** Clinical reasoning for continuing treatment in patients without pneumatosis on AUS

US Findings	Clinical Reasoning for NEC Treatment
Pneumatosis Absent	• Persistent abdominal distension with increasing pressor requirements
	• Persistent bloody stools
	• Persistent abdominal distension with episodes of bradycardia and desaturation
	• Clinical deterioration with respiratory distress requiring intubation
	• Persistent abdominal distension with metabolic acidosis
	• Clinical deterioration requiring intubation and persistent thrombocytopenia
Equivocal	• Clinical deterioration requiring intubation

**Fig. 4:**
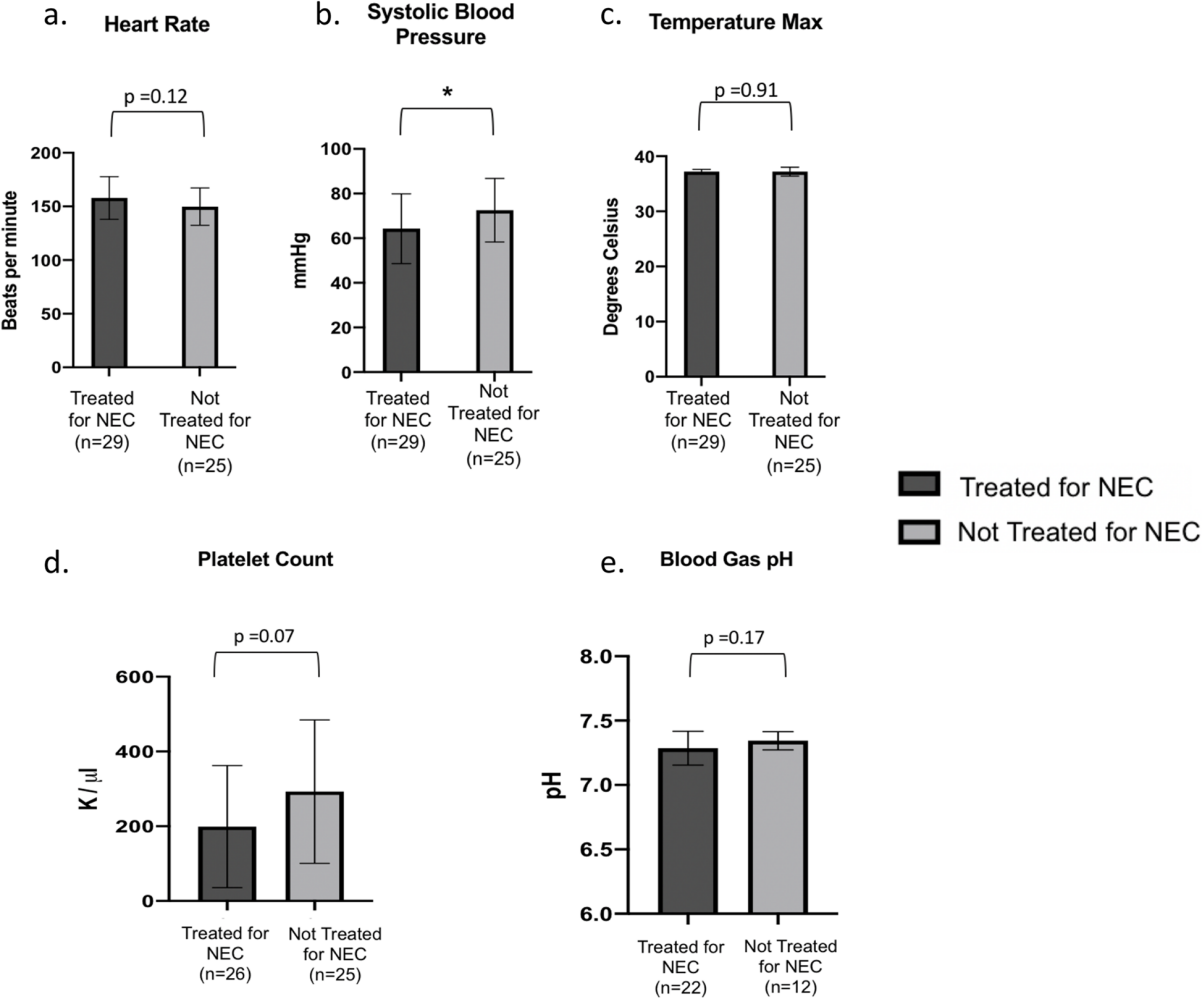
Vital Signs and Laboratory Values at Initial Evaluation. **a**-**d** Vital signs and laboratory values documented at the time of concern for NEC in treated and not treated patients. **a** Heart rate in beats per minute. **b** Systolic blood pressure in mmHg. **c** Maximum temperature in degrees Celsius. **d** Platelet count in K/μl on complete blood count (CBC) test. **e** pH on blood gas. Data is represented as mean ± standard deviation. Two-tailed Student’s t-test was utilized to calculate the difference between means. *p < 0.05

Systolic blood pressure in patients treated for NEC tended to be lower than those patients who were not treated for NEC (Fig. 4b. 64.3 mmHg vs 72.5 mmHg, respectively; *p* < 0.05). No other vital sign or laboratory value was found to differ between patients who were treated for NEC from those who were not treated (Fig. 4). Twenty-eight of the 29 patients treated for NEC (97%) had a pre-existing comorbidity. The most common comorbidity among treated patients was prematurity (86%). All 6 patients with intraventricular hemorrhage were in the NEC-treated group and 5 of the 8 patients (63%) with cardiac abnormalities were also treated for NEC.

Of the 29 patients treated for NEC, with bowel rest and IV antibiotics, 10 patients (35%) required surgical intervention including resection of affected bowel, and 2 patients (7%) were treated with intraperitoneal drain placement alone. Indications for surgical intervention listed in the medical record for the 10 patients are described in Table 3. There were 5 deaths (17%) in the treated group, 3 of these patients had required surgical intervention. The remainder of patients treated for NEC were ultimately discharged from the NICU. Patients not treated for NEC and patients receiving abbreviated courses of antibiotics based on negative AUS findings did not have recurrent issues or require treatment for NEC within a follow-up period of 7 days, and all were successfully discharged from NICU.

**Table 3:** Clinical indications for surgical intervention in patients treated for NEC

1. Persistent abdominal distention and dilated loops of bowel on AXR despite course of medical therapy	
2. Worsening abdominal distension, inability to tolerate enteral feeds, and significant thrombocytopenia	
3. New pneumoperitoneum on serial imaging	
4. Worsening thrombocytopenia and increasing ventilatory requirements	
5. Failure to progress clinically with rising bilirubin, and obstructive pattern seen on imaging with concern for stricture	
6. Clinical deterioration, fluid collection seen on AUS	
7. Persistent pneumoperitoneum despite drain placement and worsening abdominal fluid collections	
8. Worsening abdominal distension and discoloration, previously placed drain expressing succus	
9. Clinical deterioration with succus from previously placed drain	
10. Clinical deterioration with worsening thrombocytopenia	

**Fig. 5:**
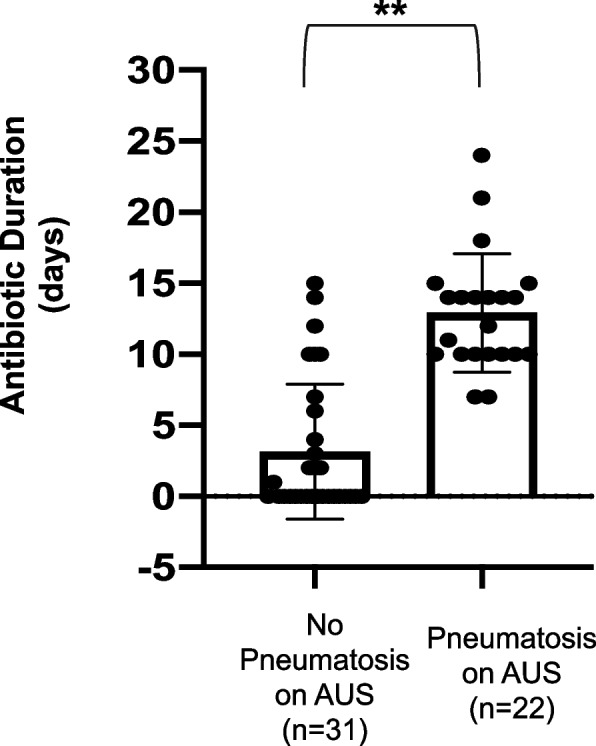
Antibiotic Duration Based on Presence or Absence of Pneumatosis on AUS. Scatter plots show individual patients. Bar graph represents mean ± standard deviation. Two-tailed Student’s t-test was utilized to calculate the difference between means. **p < 0.001

Seventeen of 31 patients (55%) with negative AUS findings were not placed on antibiotics for any duration, while all 22 of patients with pneumatosis (100%) on AUS were given antibiotic treatment (*p* < 0.001). Mean antibiotic duration for patients with pneumatosis on AUS was significantly longer than for those patients without pneumatosis on AUS (12.4 (4.7 days) vs 3.3 days (4.8 days), respectively; *p* < 0.001) (Fig. 5). Considering only patients without pneumatosis on AUS who received antibiotics, duration of antibiotics was significantly shorter than those with pneumatosis (7 (4.8 days) vs 12.4 (4.7 days), respectively; *p* < 0.001). Of the 17 patients without pneumatosis on AUS that were treated with antibiotics, 11 patients had empiric treatment discontinued once AUS confirmed absence of pneumatosis, and 6 patients were treated with full courses of antibiotics for clinically diagnosed NEC, as described previously.

## Discussion

AUS is emerging as an important radiographic adjunct in the diagnostic workup of NEC. As with many novel diagnostic modalities, its clinical utility may be easiest to utilize in cases of clinical uncertainty, particularly in cases where clinical features may raise suspicion for NEC but AXR is equivocal [8]. For this reason, in this study we specifically studied patients with equivocal AXR to see how AUS could be helpful as a diagnostic adjunct, as opposed to examining concordance between AXR and AUS, as has been described previously in the literature [8]. Radiographic findings such as pneumatosis intestinalis and portal venous gas are pathognomonic for NEC, however AXR has been shown to have a low sensitivity for such findings especially in extremely pre-term infants and in less severe cases of disease [4, 7, 8, 14, 17]. In contrast, many studies, including our own, have demonstrated that AUS may have higher sensitivity for identifying pneumatosis and portal venous gas. Using pneumatosis as a radiographic sign of NEC, we found AUS to have a sensitivity of 78.6%, a specificity of 100%, a positive predictive value of 100%, and a negative predictive value of 80.6%. These findings are congruous with other studies in the literature. In a study of 62 neonates, Faingold et al., utilized AUS to examine bowel perfusion in evaluation for NEC and found AUS to have a sensitivity of 100% and a specificity of 90%. In the same study, the sensitivity of AXR to identify free air as a sign of NEC was only 40% [12]. Similarly, Yikilmaz studied the ability of AUS to detect intestinal necrosis in infants with radiographically confirmed NEC. Sensitivity, specificity, positive predictive value, and negative predictive value were based on confirmation of intestinal necrosis at time of surgery in surgical NEC patients, and was found to be 100, 95.4, 80, and 100%, respectively [13].

Additional benefits of AUS include portability and lack of ionizing radiation, as well as ability to assess concerning bowel characteristics such as bowel wall perfusion, wall thickness, and aperistalsis [9]. In our institutional experience, an AUS examination for NEC takes only 15 to 20 min to complete and can be done bedside to avoid need for transport. On average AUS was obtained within hours of equivocal AXR, however was obtained in as little as 20 min when warranted by the clinical scenario.

While the literature shows AUS to be more sensitive and specific for diagnosing NEC, standard practice still utilizes AXR for diagnosis likely due to lack of experience and confidence of the multidisciplinary team in using this imaging modality. A European survey on imaging in NEC showed widespread agreement amongst neonatologists, pediatric surgeons, and radiologists on utilizing AXR findings for diagnosing NEC, but a reluctance among this same group to use AUS findings, particularly in institutions where clinicians had less experience with AUS [14]. Even in our institution where radiologists have a strong familiarity in utilizing AUS for diagnosis of NEC, the presence or absence of pneumatosis was the only characteristic that was consistently documented in AUS reports. Additional features of the bowel that can be evaluated via AUS that can more specifically diagnose NEC, including bowel echogenicity, bowel wall thickness, and presence of peristalsis, were not consistently noted in all AUS reads and therefore could not be accurately analyzed in this study. More consistently identifying these additional bowel features in addition to identification of pneumatosis will further increase the diagnostic accuracy of AUS in NEC.

As discussed by Tracy et al., pneumatosis, pneumoperitoneum, and bowel wall thickening were all more frequently detected by AUS than by AXR, which is consistent with other studies that have demonstrated that AUS is more sensitive for identifying signs of ischemic bowel and can identify these features at earlier time points than AXR [8]. While this raises the concern that AUS may increase the rates of false positives and lead to overtreatment, when AUS is used as an adjunctive tool in combination with clinical assessment and AXR this has not been shown to be the case. When AUS was combined with AXR, it was demonstrated by Tracy et al. that patients with an AUS negative for pneumatosis or portal venous gas had significantly shorter antibiotic courses than those with a positive study [8], findings which are concordant with those of our study. Additionally, this may make AUS an especially useful modality to rule out NEC in cases where clinical suspicion is low.

Familiarity with AXR over AUS and higher operator dependency associated with AUS may contribute to the slow integration of AUS as a diagnostic tool. To address this, at our institution we have begun to formulate a diagnostic algorithm, which includes the use of AUS in cases of in cases when AXR is equivocal or non-diagnostic. Additionally, in order to reduce operator dependency and increase consistency of examination, the pediatric radiology department at our institution has implemented a standardized approach for using and performing AUS for evaluation of suspected NEC. Establishing a diagnostic algorithm and having a standardized approach to including AUS will make the use of AUS more widespread and will likely assist with more accurate recognition of NEC and proper utilization of antibiotics. As described by Alexander et al., providing a diagnostic framework can assist in the implementation of AUS in the diagnosis of NEC in a clinical practice setting where it is not yet commonplace [9]. Future studies will focus on the effect of implementing this local algorithm on the proper diagnosis and treatment of NEC.

Most of the literature regarding AUS in NEC focuses on the imaging findings of AUS to diagnose NEC compared with AXR, and not on the use AUS as means to decrease potentially unnecessary overtreatment of the disease [1, 4, 8, 10, 12]. The impetus for this project came out of the antibiotic stewardship project at our institution, which focuses on improving the appropriate use of antibiotics to improve patient outcomes and reduce antibiotic resistance. It is becoming increasingly well known that prolonged antibiotic exposure is associated with higher odds of death and higher odds of NEC, particularly in pre-term infants [18–21]. While early signs of NEC are nonspecific and make the diagnosis difficult, establishing a diagnosis quickly and excluding patients with low suspicion for NEC limits antibiotic use in patients who do not need it. The balance between swift initiation of NEC treatment to reduce mortality and avoidance of overtreatment with antibiotics overtreatment is difficult. In this study, we found that the absence of pneumatosis on AUS impacted clinical decision making by significantly reducing unnecessary antibiotic usage and duration in this population of infants with suspected NEC and equivocal AXR findings, who historically would have received a full course of antibiotics. Additionally, all patients with pneumatosis on AUS were appropriately treated. These findings demonstrate that AUS assisted clinical decision making by either confirming or ruling-out NEC based on the presence or absence of pneumatosis.

The limitations of this study should be noted. This study is a single institutional experience which may be influenced by individual practice patterns and not entirely represent the whole population of patients being evaluated for NEC. There is also inherent selection bias in our patient sample as these were patients selected due to equivocal AXR findings and may not be representative of all patients undergoing evaluation for NEC. Given that we obtained our data from a radiology database that contained only those patients with equivocal AXRs, we do not have data on all of the AXRs done for NEC within our study period, and therefore cannot report the rate of equivocal AXRs at our institution. Future studies will examine this prospectively where each patient with concern for NEC will be cataloged along with all imaging studies obtained. As data extraction was obtained through a retrospective chart review, there may have been inconsistent documentation in the symptoms concerning for NEC and the clinical judgement used to diagnose, initiate, or continue treatment. We examined the presence of pneumatosis specifically, and no other signs of NEC on AUS, as this sign alone can positively define NEC based on the Modified Bells Criteria [3]. It should be noted that while this study focuses on the identification of pneumatosis by AUS and AXR, a combination of findings was used for final diagnosis of NEC. Ultimately, the diagnosis of NEC and decision to treat was made based on clinical judgement of the neonatology and surgical teams, and it was not formally objective or standardized.

AUS as an adjunct to AXR can aid in diagnosing NEC based on the presence of pneumatosis seen on imaging. Absence of pneumatosis on AUS is useful in informing clinical decision making and can lead to decreased use of antibiotics. While a randomized control trial is not feasible, a larger prospective evaluation on the influence of AUS and AXR findings on clinical decision making will further strengthen the data available on the use of AUS in the diagnosis of NEC. This will help facilitate more widespread use of AUS as a diagnostic adjunct in NEC evaluation.

## Data Availability

The datasets used and/or analyzed during the current study are available from the corresponding author on reasonable request.
